# Antibody-drug conjugates for the treatment of lymphoma: clinical advances and latest progress

**DOI:** 10.1186/s13045-021-01097-z

**Published:** 2021-06-05

**Authors:** Yurou Chu, Xiangxiang Zhou, Xin Wang

**Affiliations:** 1 Department of Hematology, Shandong Provincial Hospital, Cheeloo College of Medicine, Shandong University, No.324, Jingwu Road, Jinan, 250021 Shandong China; 2 Department of Hematology, Shandong Provincial Hospital Affiliated to Shandong First Medical University, Jinan, 250021 Shandong China; 3 School of Medicine, Shandong University, Jinan, 250012 Shandong China; 4 Shandong Provincial Engineering Research Center of Lymphoma, Jinan, 250021 Shandong China; 5 Branch of National Clinical Research Center for Hematologic Diseases, Jinan, 250021 Shandong China; 6 National Clinical Research Center for Hematologic Diseases, the First Affiliated Hospital of Soochow University, Suzhou, 251006 China

**Keywords:** Antibody-drug conjugates, Lymphoma, Immunotherapy, Clinical trials

## Abstract

Antibody-drug conjugates (ADCs) are a promising class of immunotherapies with the potential to specifically target tumor cells and ameliorate the therapeutic index of cytotoxic drugs. ADCs comprise monoclonal antibodies, cytotoxic payloads with inherent antitumor activity, and specialized linkers connecting the two. In recent years, three ADCs, brentuximab vedotin, polatuzumab vedotin, and loncastuximab tesirine, have been approved and are already establishing their place in lymphoma treatment. As the efficacy and safety of ADCs have moved in synchrony with advances in their design, a plethora of novel ADCs have garnered growing interest as treatments. In this review, we provide an overview of the essential elements of ADC strategies in lymphoma and elucidate the up-to-date progress, current challenges, and novel targets of ADCs in this rapidly evolving field.

## Introduction

**Fig. 1:**
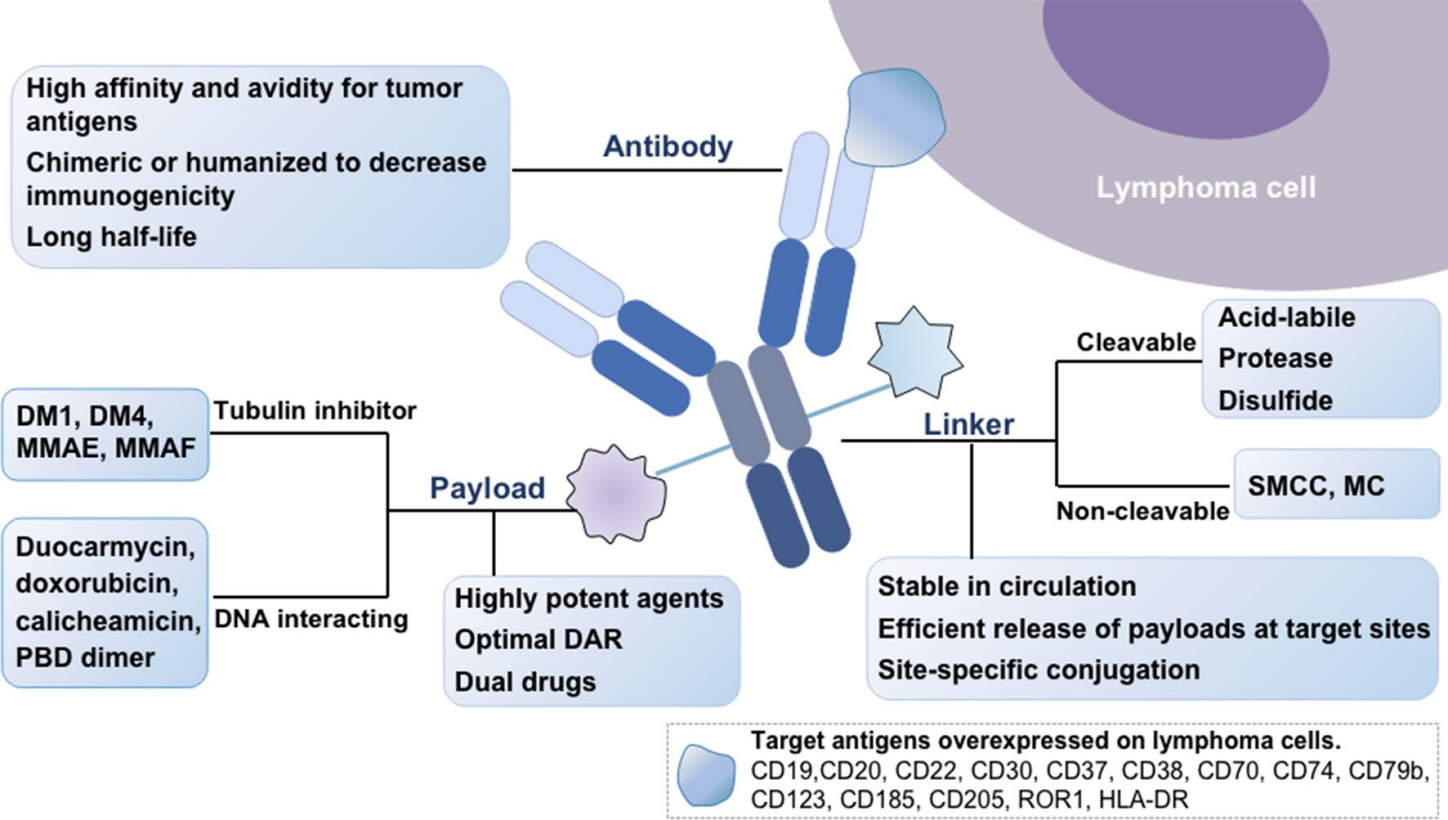
Structure and properties of an antibody-drug conjugate. MMAE: monomethyl auristatin E; MMAF: monomethyl auristatin F; DM1: N20-deacetyl-N20-(3-mercapto-1-oxopropyl)-maytansine; DM4: N20-deacetyl-N20-(4-mercapto-4-methyl-1-oxopentyl)-maytansine; MC: maleimidocaproyl; SMCC: succinimidyl-4-(*N*-maleimidomethyl)-cyclohexane-1-carboxylate; PBD: pyrrolobenzodiazepines

Chemotherapy represents an essential pillar for the treatment of various forms of hematological malignancies. However, a common theme emerges where these chemotherapy agents are usually associated with nonspecific toxicities and increasing drug resistance, presumably because of their high potency but low tumor selectivity. In this respect, monoclonal antibodies (mAbs) such as rituximab and obinutuzumab designed to specifically bind an antigen on a cancerous cell have been proven to play an essential role in lymphoma treatment [1–4]. With the development of mAb technologies, antibody–drug conjugates (ADCs), comprising a mAb connected to a small molecule cytotoxic payload via a covalent linker (Fig. 1), have emerged as a novel class of promising immunotherapies in lymphoma.

**Fig. 2:**
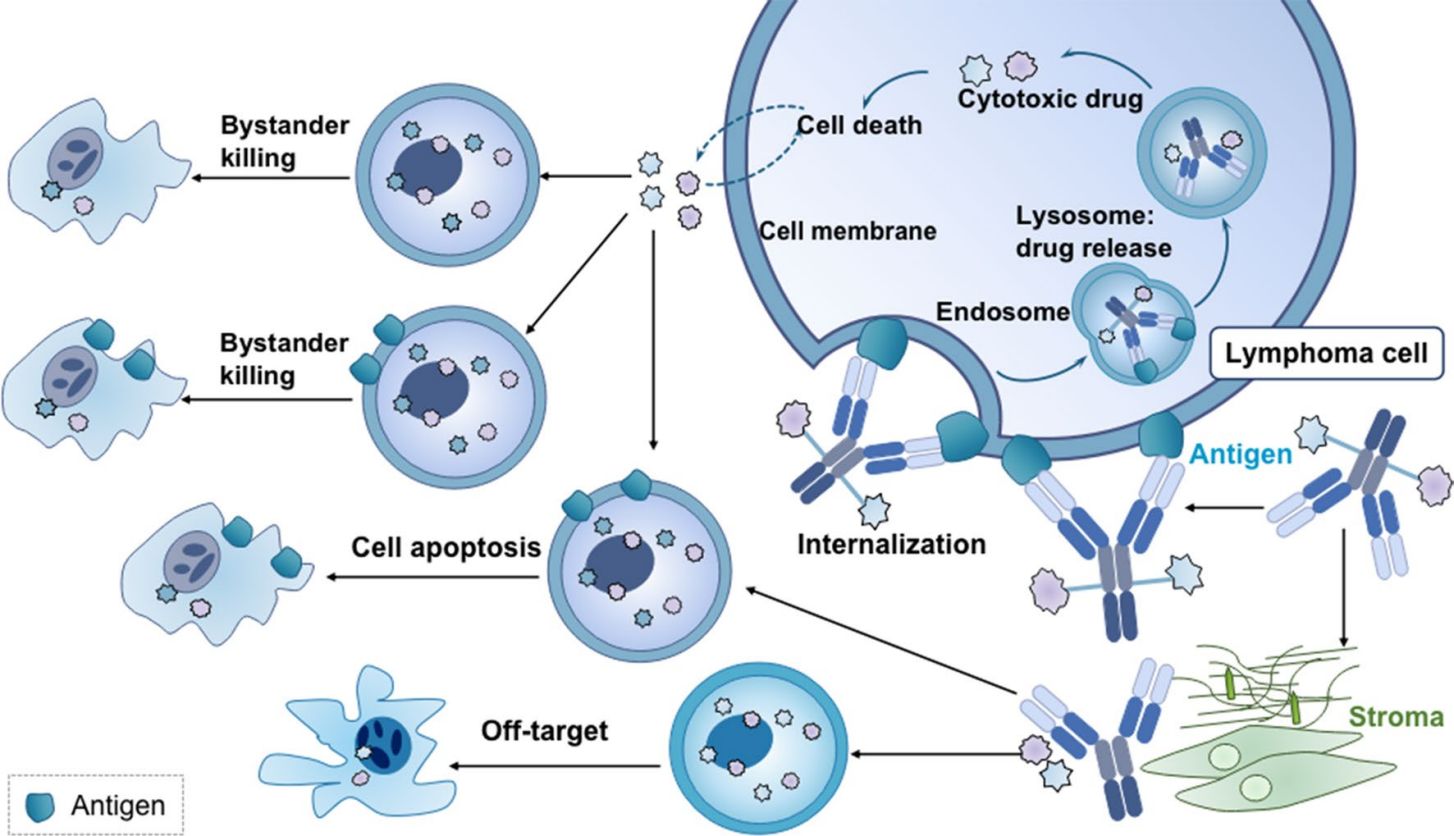
ADC mechanisms of action. Once bound to the corresponding cell-surface antigen of cancer cells, the ADC is internalized, and the cytotoxic payload is released, resulting in cell apoptosis. The drug can also diffuse into adjacent cells regardless of antigen positivity, resulting in cell death, termed “bystander killing”

Once attached to the corresponding cell-surface antigen of cancer cells, the ADC is internalized, and the cytotoxic payload is released, causing cell cycle termination and cell apoptosis. The drug can also diffuse into adjacent cells even if the cells are target-negative, resulting in cell death termed “bystander killing” (Fig. 2). This effect is generally believed to occur following the process of surface antigen targeting and internalization, but it was recently suggested to occur independently of internalization [5]. Despite the relatively straightforward molecular platform of ADCs, their application in clinical practice is hampered by multiple factors, including the narrow therapeutic index, the selection of the corresponding antibodies, the stability of the linkers, and the internalization rate of the payloads [6]. Hence, the generation of an efficacious and highly stable ADC is dependent on the proper arrangement of all sections.

At present, three ADCs, brentuximab vedotin (BV), polatuzumab vedotin (Pola), and loncastuximab tesirine, have been approved by the Food and Drug Administration (FDA) for various types of lymphoma, and these agents exert favorable effects in clinical applications [7–10]. Furthermore, additional ADCs are presently in clinical trials to evaluate whether they can be efficacious treatment options for patients in diverse clinical settings. The topics discussed here involve the fundamental components of ADC strategies and focus on exploring opportunities for previously incurable subjects and summarizing the ADCs currently in clinical use and development (Table 1).

**Table 1:** Antibody-drug conjugates in clinical trials for lymphoma

ADC	Target	Linker	Payload	Indication	Result	Trial
*ADCs approved for lymphoma*						
Brentuximab vedotin	CD30	Val-Cit	MMAE	R/R HL	ORR 75%, CR 34%	phase II [48]
				R/R HL	ORR 54.4%, median PFS 8.2 months	phase III [49]
				R/R HL	ORR 75%, CR 42%	phase I [51]
				Post-ASCT consolidation for HL	Median PFS 42.9 months, 5-year PFS 59%	phase III [52]
				Post-ASCT consolidation for HL	18-month PFS 95%, 18-month OS 98%	phase II [53]
				Salvage therapy for R/R HL	2-year OS 95%, 2-year PFS 74%	phase II [54]
				Salvage therapy for R/R HL	ORR 84%, CR 79%	phase II [55]
				Salvage therapy for R/R HL	ORR 85%, CR 67%, 2-year PFS 78%	phase I/II [56]
				Untreated HL	ORR 86%, CR 73%, 5-year PFS 82%	phase III [58]
				Untreated HL	ORR 61%, CR 48%	phase II [59]
				R/R ALCL and R/R MF	ORR4 56.3%, CR 16%, median PFS 17.2 months	phase III [60]
				R/R CTCL and R/R PTCL	ORR 38%, CR 17%	phase II [61]
				Untreated PTCL	ORR 83%, CR 68%, 5-year PFS 51%	phase III [63]
				Untreated ALCL	2-year EFS 79%, 2-year OS 97%	phase II [64]
				R/R PMBCL and R/R mediastinal GZL	PMBCL: ORR 73%, CR 37% Mediastinal GZL: ORR 70%, CR 50%	phase I/II [67]
				Untreated PMBCL, DLBCL, and GZL	ORR 100%, CR 86%, 2-year PFS 85%,	phase I/II [68]
Polatuzumb vedotin	CD79b	Val-Cit	MMAE	R/R DLBCL and R/R FL	FL: ORR 70%, CR 45%; DLBCL: ORR 54%, CR 21%	phase II [71]
				R/R DLBCL	ORR 45%, CR 40%, median OS 12.4 months,	phase II [25]
				R/R DLBCL	ORR 65%, CR 31%, median DOR 5.8 months	phase I/II [72]
				R/R FL	ORR 76%, CR 65%	phase I/II [73]
				Untreated DLBCL	ORR 89%, CR 77%	phase I/II [74]
Loncastuximab tesirine	CD19	Val-Ala	PBD dimer	R/R NHL	ORR 46%, CR 27%, median DOR 5.4 months	phase I [76]
				R/R DLBCL	ORR 48%, CR 24%, median DOR 10.3 months	phase II [78]
ADCs explored in clinical settings						
Coltuximab ravtansine	CD19	SPDB	DM4	R/R DLBCL	ORR 44%, CR 15%	phase II [79]
				R/R DLBCL	ORR 31%	phase II [81]
SGN-CD19A	CD19	MC	MMAF	R/R B-NHL	ORR 33%, CR22%, median DOR 40 weeks	phase I [82]
MT-3724	CD20	/	SLTA	R/R DLBCL	ORR 30%, CR 10%	phase I [100]
Inotuzumab ozogamicin	CD22	Acid-labile	Calicheamicin	R/R NHL	FL: ORR 87%, 2-year PFS 68% DLBCL: ORR 74%, 2-year PFS 42%	phase III [88]
				R/R NHL	ORR 41%, 18-month OS 35%	phase III [89]
				R/R NHL	ORR 53%, CR 20%	phase I [90]
				R/R B-NHL	ORR 84%, CR 24%	phase I [91]
				R/R indolent B-NHL	ORR 67%, CR 31%, median PFS 12.7 months	phase II [92]
Pinatuzumab vedotin	CD22	Protease-cleavable	MMAE	R/R DLBCL and R/R indolent NHL	DLBCL: ORR 36%, Indolent NHL: ORR 50%	phase I [96]
				R/R DLBCL and R/R FL	DLBCL: ORR 60%, CR 26%; FL: ORR 62%, CR 5%	phase II [71]
TRPH-222	CD22	Non-cleavable	Maytansinoid	R/R NHL	ORR 27.2%, CR 22.7%	phase I [97]
				R/R HL	ORR 69%, CR 44%, median DOR 7.7 months, median PFS 6.7 months	phase I [109]
IMGN529	CD37	SMCC	DM1	R/R B-NHL	ORR 13%	phase I [113]
AGS67E	CD37	Val-Cit	MMAE	R/R NHL	ORR 22%, CR 14%	phase I [116]
STRO-001	CD74	Non-cleavable	Maytansinoid	Advanced B cell malignancies	ORR 25%, CR 6.3%	phase I [125]
Iladatuzumab vedotin	CD79b	Protease-cleavable	MMAE	R/R B-NHL	ORR 85%; DLBCL: ORR 60%, CR 43%	phase I [128]
VLS-101	ROR1	UC-961	MMAE	R/R NHL	MCL: ORR 47%, CR 20%; DLBCL: ORR 80%, CR 40%	phase I [131]

ADC, antibody-drug conjugate; ASCT, autologous stem cell transplantation; ALCL, anaplastic large cell lymphoma; CTCL, cutaneous T cell lymphoma; HL, Hodgkin lymphoma; NHL, non-Hodgkin lymphoma; R/R, relapsed or refractory; CR, complete  response; DLBCL, diffuse large B cell lymphoma; DOR, duration of response; FL, follicular lymphoma; MCL, mantle cell lymphoma; GZL, gray zone lymphoma; CLL, chronic lymphocytic leukemia; PTCL, peripheral T cell lymphoma; MF, mycosis fungoides; ORR, overall response rate; OS, overall survival; PFS, progression-free survival; EFS, event-free survival; ORR4, ORR lasting at least 4 months

## Engineering ADCs

### Antigens and corresponding antibodies

Theoretically, an ideal antigen is broadly and homogeneously expressed on malignant cells and hardly present on normal cells, thereby maximizing efficacy and minimizing systemic toxicity. In most cases, ADCs are taken up by selective internalization via receptor-mediated endocytosis and yield intracellular cytotoxicity. The expression of homogeneous target antigens is not absolutely required for ADC efficiency, as heterogeneous tumors may benefit from bystander killing, which can affect proximally located antigen-negative tumor cells [11]. The effectiveness of the ADC-mediated bystander effect mostly relies on factors such as the extent of ADC internalization, the type of linkers, and the physicochemical properties of the attached payloads [5].

Antibodies used for ADCs for lymphoma are made to be chimeric, humanized, or fully human to generate the best immunogenicity profile. Owing to its favorable stability, immunoglobulin G (IgG) is the predominant class used in ADCs, and IgG1 represents a major subtype in the clinic [12]. IgG can induce antibody-dependent cell-mediated cytotoxicity (ADCC), activation of antibody-dependent cell-mediated phagocytosis (ADCP), and the complement pathway by triggering FcγR-expressing cells [13]. Nevertheless, the strong induction of these effects may maximize tumor uptake at the expense of unacceptable systemic toxicity. The IgG4 isotype and Fc-mutated variants of the IgG1 isotype are designed to attenuate toxicity [14]. Non-IgG binding proteins may be a substitute that can be tailored for individual applications with varying degrees of stability and optimized efficacy [15, 16]. The IgM type has also been considered in development because of its greater number of engineered functional sites and higher drug-antibody ratio (DAR) [17]. In addition, triple variable domain Fab, with a small molecular weight, has been proposed as a new format that contributes to deeper tumor tissue distribution, affording stable generation of ADCs [18]. Nevertheless, the design of antibody formats needs to strike a favorable balance between reduced molecular weight and decreased plasma half-life, which can facilitate and vitiate tumor uptake, respectively.

### Alternative payloads

The cytotoxic payloads, as the final effector component, should meet core requirements, such as having strong potency in the minute range within which they are released. The payloads that have been validated in the clinic are categorized into two major groups, namely, DNA-interacting agents (e.g., duocarmycin, calicheamicin, doxorubicin, camptothecin analogs, and pyrrolobenzodiazepine [PBD] dimers) or tubulin inhibitors (e.g., maytansines and auristatins). Inotuzumab ozogamicin (InO) employs calicheamicin as an efficient payload and has been investigated in multiple lymphoid malignancies, including diffuse large B cell lymphoma (DLBCL), mantle cell lymphoma (MCL), and follicular lymphoma (FL) [19–21]. PBD dimers target DNA minor grooves and therefore cause DNA alkylation. ADCT-301 and ADCT-402, which deliver PBD dimers, are under development [22, 23].

Tubulin inactivators, the auristatins monomethyl auristatin E (MMAE), and monomethyl auristatin F (MMAF) impede microtubule polymerization, leading to cell apoptosis. MMAE is employed in Pola and BV [24, 25], and MMAF is utilized in denintuzumab mafodotin [26]. In addition, MMAE, unlike MMAF, yields bystander effect in vivo [27]. Maytansine derivatives are mainly divided into two categories: DM1 and DM4. DM1 agents are highly potent maytansinoids (emtansine and mertansine) that have broad killing effects on non-Hodgkin lymphoma (NHL) xenografts in vivo [28]. DM4 agents comprise soravtansine and ravtansine (e.g., coltuximab ravtansine), which can augment the bystander effect of adjacent cells in vivo, thereby eradicating tumors [29, 30].

Compared with anti-microtubule payloads, DNA-damaging agents can provide higher potency and enable ADCs to target less abundant tumor antigens [31]. A trend of assessing DNA-interacting agents as ADC payloads has been emerging [32, 33]. Indolinobenzodiazepine dimers (termed IGNs), a novel chemical class of DNA-interfering payloads, feature enhanced bystander effect and tolerability as well as expanded therapeutic utility [34]. Seco-CBI dimers, a class of duocarmycin analogs, are presently being explored in NHL and are conjugated to anti-CD22 mAb, affording a more durable response and higher activity in indolent NHL [35].

### Linkers and site-specific conjugation

Linkers provide a functional handle for efficient conjugation to antibodies. At present, cleavable and non-cleavable linkers are two major classes widely applied in the clinical setting. Due to their ability to liberate diffusible payloads, cleavable linkers are implemented in a broad range of tumor types. Three main types of cleavable linkers respond to physiological stimuli, such as low pH: acid-labile hydrazone linkers, disulfide linkers, and protease-cleavable linkers. *N*-succinimidyl-4-(2-pyridyldithio)-pentanoate (SPP) and *N*-succinimidyl-4-(2-pyridylthio)-butanoate (SPDB) represent two classes of disulfide linkers that are unstable at high glutathione concentrations (e.g., used in the maytansinoid-based ADC naratuximab emtansine). Protease-cleavable linkers, the most stable type, comprise dipeptide sequences such as valine-citrulline (Val-Cit) and valine-alanine (Val-Ala) that are generally cleaved by cathepsin B intracellularly (e.g., that used in Pola). Nonetheless, sufficient extracellular proteolytic cleavage of the Val-Cit linker is also observed, indicating that endocytosis of ADC is not required under certain conditions and that developing non-internalizing antibodies may be a potential option for increasing antitumor activity [36].

Concerning the pursuit of increased plasma stability, non-cleavable linkers appear to be a better option for ADCs. Non-cleavable linkers mainly depend on sufficient processing of mAbs within the lysosome after ADC internalization to release the toxin due to the lack of cell permeability. These linkers are accompanied by limited bystander effect and have lower membrane permeability.

The attachment between mAb and linker is a crucial parameter of ADCs as it determines the drug-to-antibody ratio (DAR) and consequently the stability, efficacy, and homogeneity of the ADCs. Site-specific methods are proposed to optimize the DAR and maintain favorable pharmacokinetics while maximizing payloads in the development of ADCs. Several site-specific conjugation approaches that utilize integrated non-natural amino acids, engineered cysteine residues [37] or enzymatic modifications, including transglutaminases [38], sortase A [39], glycosyltransferase [40] and formylglycine-generating enzyme [41], have been reported. The chemoenzymatic approach can harvest a high yield of ADCs with suitable DARs and attractive conjugation efficiency by achieving site-specific conjugation and generating precisely controlled modification sites [42].

The site-specific method has enabled the generation of multivalent conjugated drugs. For example, a heterotrifunctional linker was designed to prepare a dual-cytotoxic (MMAE and DM1) drug conjugate in a site-specific manner, highlighting the potential of ADCs with distinct mechanisms of action for targeting neoplasms and attaining obvious synergy compared with single agents [43]. Additionally, dual-drug combinations also offer possibilities for circumventing ADC-related drug resistance in practice [44]. Therefore, selection and design strategies are complementary and particularly powerful when used in combination.

## ADCs approved for lymphoma

### Brentuximab vedotin

CD30, a transmembrane glycoprotein belonging to the tumor necrosis factor (TNF) receptor superfamily, is expressed on a small subset of activated B and T lymphocytes and restricted to normal tissues. CD30 is of high interest as a therapeutic target for antibody-based treatments owing to its excessive and selective expression on cancer cells [45]. The prognostic and therapeutic impacts of CD30 expression have been investigated in Hodgkin lymphoma (HL), anaplastic large cell lymphoma (ALCL), cutaneous T cell lymphoma (CTCL), and primary mediastinal B cell lymphoma (PMBCL) [7, 46].

BV comprises a CD30 targeting chimeric IgG1 mAb, an MMAE payload moiety, and a Val-Cit linker. A series of studies have explored the clinical benefits of BV in diverse settings, such as second-line therapy for relapsed or refractory (R/R) disease, consolidation therapy, salvage therapy, and frontline therapy in HL (Table 1). The encouraging results led to the approval of BV by the U.S. FDA for patients with relapsed classical Hodgkin lymphoma (cHL) and relapsed ALCL [47]. In a pivotal phase II study of BV monotherapy, the overall response rate (ORR) was 75%, with 34% complete response (CR) in patients with R/R HL who failed autologous stem cell transplantation (ASCT) [48]. For patients achieving a CR, the median duration of response (DOR) was 20.5 months, with 5-year progression-free survival (PFS) and overall survival (OS) rates of 52% and 64%, respectively. The most common side effects related to the drug were peripheral neuropathy (PN), nausea, fatigue, neutropenia, and diarrhea. In the phase III KEYNOTE-204 study (NCT02684292) involving patients with R/R cHL who had received more than two prior therapies and were treated with BV or pembrolizumab (a PD-1 inhibitor) monotherapy, the ORR was 54% for the BV arm and 65% for the pembrolizumab arm [49]. The reported median PFS was 8.2 months for BV and 12.6 months for pembrolizumab. For patients experiencing severe adverse effects (AEs), the proportion was comparable in the BV and pembrolizumab arms (24% vs. 23%). This trial suggested the potential benefit of BV and pembrolizumab combination therapy in patients who are heavily pretreated. Nonetheless, some patients will ultimately develop BV resistance and may partially attribute to the upregulation of NF-kappaB [50] or multidrug resistance pump (MDR1) [51]. The combination therapy of BV and cyclosporine A (an MDR1 inhibitor) was recently examined in a phase I trial (NCT03013933) to combat BV resistance in cHL, which reported an ORR of 75%, a CR rate of 42%, and a modest toxicity profile [51].

The phase III AETHERA trial (NCT01100502) measured the efficacy and safety profiles of BV in patients with cHL in the post-transplant consolidation setting [52]. The 5-year PFS rates were 59% in patients with BV and 43% in patients with placebo. The most common AEs associated with the therapeutic agent included neurotoxicity (67%), infection (60%), and neutropenia (35%). The majority of PN and neutropenia cases were reversible and managed with dose delay or reduction. Recently, a phase II study evaluated the results of 59 participants with R/R aggressive HL receiving BV and nivolumab (Nivo), a PD-1 inhibitor, as post-ASCT consolidation therapy [53]. The estimated 18-month OS and PFS rates were 98% and 95%, respectively. Treatment was well tolerated, with common AEs of PN (51%) and neutropenia (42%). The encouraging results suggested that BV plus Nivo  may provide prolonged remission in patients at an advanced stage.

The incorporation of BV in salvage therapy for patients with R/R diseases has been evaluated by a few studies. One phase II study (NCT02280993) including 55 R/R cHL patients treated with BV plus dexamethasone, cisplatin, and cytarabine (DHAP) with a PFS rate of 74% and OS rate of 95% at the 2-year follow-up was recently reported [54]. For patients who proceeded to ASCT, 42 patients achieved a metabolic CR and 5 achieved a metabolic partial response (PR). A BV and bendamustine regimen also provided a robust efficacy benefit in HL patients in a single-arm multicenter phase II study [55]. The ORR was 84%, with 30 patients (79%) achieving a CR and 2 patients (5%) achieving a PR. In addition, 33 patients underwent ASCT. The estimated 3-year OS and PFS rates were also promising, at 88% and 67%, respectively. Skin reactions were rather frequent (65%) and should be monitored during treatment. Recently, BV plus Nivo therapy, the first chemo-free combination, was reported to have an ORR of 85% and a CR rate of 67% for all treated patients with cHL in a phase I/II trial (NCT02572167) [56]. The estimated 2-year PFS rate in all enrolled patients was 78%. For patients who proceeded to ASCT after receiving BV + Nivo, the proportion was 91%, suggesting that further exploration of this combination treatment as a bridge to ASCT in a larger cohort is warranted.

The approval for frontline use in combination with chemotherapy was based on the impressive activity and manageable safety profiles observed in the phase III ECHELON-1 trial in advanced-stage cHL [57]. Updated data from the ECHELON-1 cohort (NCT01712490) demonstrated an overall risk–benefit ratio favoring the BV plus doxorubicin, vinblastine, and dacarbazine (A + AVD) arm over the doxorubicin, bleomycin, vinblastine, and dacarbazine (ABVD) arm in patients with previously untreated cHL irrespective of prognostic risk score [58]. At a 5-year follow-up, a durable efficacy benefit was found in participants receiving A + AVD compared with ABVD, with an improved PFS rate (82% vs. 75%), albeit with a comparable ORR (86% vs. 83%) and CR rate (73% vs. 70%). A higher incidence of PN was seen in the A + AVD (67% vs. 43%) arm with an increased rate of febrile neutropenia (19% vs. 8%) and a decreased rate of pulmonary-related toxicity (2% vs 7%). ABVD therapy was considered unsuitable for further development based on a relatively high incidence of pulmonary-related toxicity. In a single-arm, phase II trial (NCT02758717), elderly patients who were considered unsuitable for standard chemotherapies received BV + Nivo with an ORR of 61% and a CR rate of 48% [59]. Neurotoxicity was noted in 14 (30%) of 46 patients and may have accounted for dose adjustments. Currently, a phase III trial (NCT03907488) of the A + AVD arm versus the Nivo + AVD arm is undergoing in patients with newly diagnosed advanced-stage HL.

BV has been evaluated in various types of R/R NHL, including mycosis fungoides (MF) and primary cutaneous ALCL. Efficacy analysis was conducted in a pooled population of 128 patients with CD30-positive MF or primary cutaneous ALCL who failed prior systemic therapies and were enrolled in the multicenter randomized phase III trial (ALCANZA) [60]. ORR lasting at least 4 months (ORR4) intensely favored the BV arm compared to the control (methotrexate or bexarotene) arm (56.3% vs. 12.5%). Notably, this favorable clinical efficacy of the BV group was observed for other endpoints, such as CR rate (16% vs. 2%) and median PFS (17.2 vs. 3.5 months), and manifested as a reduction in tumor burden. Grade 3 to 4 adverse events were reported less frequently in the BV group than in the control group (41% vs. 47%), while PN was seen frequently in the BV group (67% vs. 6%), most of which were grade 1 to 2. Studies combining BV with other immunomodulatory agents or chemotherapies have revealed improvements in outcomes, compared with BV monotherapy. The initial outcomes of a phase II study of BV and lenalidomide (Len) in heavily pretreated subjects with R/R CTCL and R/R PTCL reported a CR rate of 17%, a PR rate of 25%, and an ORR of 38% [61]. Recruitment of both CTCL and PTCL patients for this trial is ongoing (NCT03409432). BV has also been shown to be effective in patients with newly diagnosed PTCL. The ECHELON-2 trial (NCT01777152) observed a consistent response and an OS benefit in aggressive PTCL (predominantly ALCL) with the BV, cyclophosphamide, doxorubicin, and prednisone (A + CHP) regimen compared with a proven standard therapy (CHOP) [62]. The A + CHP group had a superior 5-year PFS rate (51% vs. 43%), an improved CR rate (68% vs. 56%) and ORR (83% vs. 72%), and slightly increased toxicity [63]. The addition of BV in frontline therapy for ALCL has also been evaluated in several studies in the hope of improving cure rates. A recently reported phase II trial enrolled 68 pediatric patients with newly diagnosed ALCL and treated them with BV in combination with standard chemotherapy [64]. The 2-year event-free survival (EFS) rate was 79%, and the 2-year OS rate was 97%, with no cases of severe neuropathy occurred, suggesting that the addition of BV to standard chemotherapies may be tolerable and used to prevent relapses in children.

Additionally, the BV plus Len regimen has also been explored in patients with R/R DLBCL who relapsed after hematopoietic stem cell transplantation (HSCT) and were in need of novel therapies. Data from a phase I trial showed an ORR of 57% in 37 subjects and an ORR of 73% in the CD30-positive subgroup (*n* = 15) [65]. The encouraging results of BV plus Len regimen led to assessments of efficacy in combination with Len and rituximab versus placebo in combination with Len and rituximab in a phase III study (NCT04404283) for patients with R/R DLBCL. Moreover, a combination with immune checkpoint inhibitors could be a feasible option for R/R B-NHL [66]. The BV plus Nivo regimen has shown activity in patients with R/R PMBCL who have received at least two prior therapies, and exhibited advantages over the use of PD-1 inhibitor or BV alone [7]. Efficacy analysis of 30 patients in the phase I/II study (NCT02581631) reported an ORR of 73%, with 37% of patients achieving a CR. Notable treatment-emergent adverse events (TEAEs) included PN (10%) and thrombocytopenia (10%). Three patients discontinued treatment owing to severe PN. The updated data of the trial showed an ORR of 70% (50% CR) and a tolerable safety profile in 10 patients with R/R mediastinal gray zone lymphoma (GZL) receiving BV + Nivo [67]. The time to CR was 1.2 months and 5 patients who achieved a CR were bridged to HSCT. Based on the high CR rate, this regimen may provide an alternate option for bridging to hematopoietic cell transplantation. Furthermore, the BV combined with rituximab, cyclophosphamide, doxorubicin, and prednisone (R-CHP) regimen was also assessed as frontline therapy in a phase I/II multicenter trial (NCT01994850) using 6 cycles of treatment to assess toxicity and efficacy for 29 patients with CD30-positive B cell lymphomas, including 22 PMBCL, 5 DLBCL, and 2 GZL [68]. After systemic treatment was administered, the ORR was 100%, with 86% of patients achieving a CR by the time therapy was completed. The 2-year PFS and OS rates were 85% and 100%, respectively. Subtype analysis revealed that the 2-year PFS rate in patients with PMBCL was 86%. The treatment may provide durable responses for patients with CD30-positive B cell lymphomas and warrants further exploration. Thus, BV-based therapies may be efficacious and safe treatment options for patients at a high risk of relapse.

### Polatuzumab vedotin

CD79b is a component of the B-cell receptor (BCR) complex required for proper cellular localization, trafficking, and signal transduction [69]. CD79b is restricted to B cells and highly prevalent in B cell leukemia and lymphoma with minimal expression in normal tissue, appearing ideally suited to serve as the targeting moiety [70].

The Pola structure contains an anti-CD79b mAb site-specifically coupled with MMAE through a Val-Cit linker similar to the structure of the anti-CD22 ADC pinatuzumab vedotin (Pina). The multicenter, phase II ROMULUS study (NCT01691898) illustrated that rituximab plus Pola (R-pola) showed significant benefit over rituximab plus Pina (R-pina) in patients with R/R DLBCL and R/R FL, and both arms compared favorably with other rituximab-based immunochemotherapy arms [71]. Regardless of CD79b or CD22 expression level, R-pola was related to a more favorable outcome in the FL subgroup, including an ORR of 70% (45% CR) and a median PFS of 15.3 months. In DLBCL patients, the R-pola arm showed a comparable ORR (54% vs. 60%) and CR rate (21% vs. 26%) with the control arm. In addition, R-pola was associated with fewer grade 3 or higher side effects in both the DLBCL and FL cohorts.

Currently, there is no standard treatment strategy for some patients with R/R DLBCL who are transplant-ineligible and for those who relapse after HSCT. In a multicenter phase II trial (NCT02257567), patients were randomly assigned to the Pola plus bendamustine and rituximab (pola-BR) arm or bendamustine plus rituximab (BR) arm. The results showed that compared with the BR group, the ORR (45% vs. 18%), CR rate (40% vs. 17.5%), median OS (12.4 vs. 4.7 months), and PFS (9.5 vs. 3.7 months) in the pola-BR arm were significantly improved, and the AEs were acceptable [25]. Another phase Ib/II trial (NCT02611323) investigated the efficacy and tolerability of a novel triplet combination (Pola, venetoclax, and rituximab) in 57 participants with R/R DLBCL [72]. The reported ORR and CR rate were 65% and 31%, respectively, with a median DOR of 5.8 months. Notable AEs causing dose reduction or interruption of any drug occurred in 18% and 61% of patients, respectively. Further evaluation of this combination is warranted due to the limited number of patients recruited. Based on the aforementioned data, Pola is also under exploration in patients with R/R MCL in an ongoing phase II trial (NCT04659044) that is recruiting patients receiving the same combination therapy of Pola, venetoclax, and rituximab.

The benefit of adding Pola in combination with other immunotherapies has been explored in a few studies. A phase Ib/II study (NCT02600897) sought to determine the efficacy and safety profiles of a triplet combination of Pola, obinutuzumab (G), and lenalidomide (Pola-G-Len) in patients with R/R FL [73]. The ORR was 76%, with a CR rate of 65% in the primary efficacy population (*n* = 46). Of those patients who were refractory to their last treatment, 71% achieved a CR. Grade 3 or worse adverse events included neutropenia (50%), thrombocytopenia (23%), infections (16%), and anemia (14%). TEAEs causing a dose reduction or cycle cessation arose in 19 (34%) and 41 (73%) patients, respectively, and the majority were attributed to intolerability of Len. A more extended period of follow-up, through and beyond maintenance treatment, is ongoing. Despite the desirable antitumor activity of triplet therapy, optimization of doses or prophylactic use of granulocyte colony stimulating factor (G-CSF) is needed to reduce the incidence of AEs.

An open-label, non-randomized phase Ib/II study (NCT01992653) focused on incorporating Pola into the R-CHP or G-CHP regimen as one of the frontline treatments in adult patients with DLBCL, the desirable outcomes of which contributed to FDA approval [74]. Among patients receiving Pola in combination with chemotherapy, an encouraging response with an ORR of 89% (77% CR) was achieved. Instances of severe myelosuppression included neutropenia (30%), febrile neutropenia (18%), and thrombocytopenia (9%). In light of the encouraging data mentioned above, a phase III, double-blind POLARIX trial (NCT03274492) is undergoing to investigate whether Pola combination therapy (Pola-R-CHP) can be clinically advantageous in certain DLBCL subtypes. In addition, Pola-R-CHP has shown a more favorable efficacy than the proven therapy (R-CHOP) as the frontline regimen for patients with previously untreated DLBCL. Other uncompleted phase I/II studies have investigated various types of lymphomas, such as newly diagnosed double- or triple-hit lymphoma (NCT04479267) and untreated aggressive B cell lymphoma (NCT04231877).

### Loncastuximab tesirine

CD19 is a 95 kDa glycoprotein that is critically involved in the processes of B cell proliferation, differentiation, activation, and antibody production, and it can also promote BCR signal transduction. As a biomarker, CD19 is prevalently expressed in B cell malignancies and is thought to be the most reliable surface biomarker for B cells [75].

Loncastuximab tesirine (ADCT-402), which was recently approved for use in patients with R/R large B cell lymphoma, comprises a humanized mAb specifically directed to CD19, a PBD dimer, and a cleavable disulfide-bond linker. The results of 183 patients with R/R NHL who received loncastuximab tesirine in the phase I dose-expansion study (NCT02669017) reported an ORR of 46% and a CR rate of 27% with a median DOR of 5.4 months [76]. Among patients in the DLBCL, MCL, and FL subgroups, the ORRs were 42%, 47%, and 79%, respectively. Notably, the ORR was 56% in the subgroup of elderly patients (≥ 75 years old) with DLBCL, indicating encouraging efficacy. The patients tolerated the therapy well because the treatment-related toxicities were largely hematologic and generally manageable with dose delays, followed by fatigue, nausea, edema, and hepatotoxicity, while dose-limiting toxicities (DLTs) were reported in only 4 patients. The rapid onset of response also supported the further development of loncastuximab tesirine when compared with the new combination therapy of tafasitamab plus Len in patients with R/R DLBCL (1.5 vs. 2.0 months) [77]. Given the small number of patients in the FL subgroup (*n* = 14), larger randomized studies are needed for validation. The updated analysis of data from a phase II trial (NCT03589469) reported an ORR of 48%, a CR rate of 24% and a median DOR of 10.3 months in 145 evaluable patients with R/R DLBCL with a 26% frequency of severe neutropenia and a 17% frequency of grade 3 to 4 glutamyltransferase (GGT) increase [78]. The data also revealed an ORR of 46% among patients who failed prior CD19-directed chimeric antigen receptor T cell (CAR-T) therapy, indicating that these immunotargeted agents may be used consequentially in high-risk patients. A survival advantage was also noted in patients with double- or triple-hit DLBCL, with an ORR of 33% (notably all CRs) and a median DOR of 13.4 months. Other phase I trials have explored loncastuximab tesirine as part of immunochemotherapy, such as with rituximab (NCT04384484) or ibrutinib.

## ADCs explored in various clinical and preclinical settings

### Anti-CD19 ADCs

#### Coltuximab ravtansine

Coltuximab ravtansine (SAR3419) comprises DM4, a potent tubulin inhibitor, coupled to a CD19-targeting mAb through an SPDB linker [79]. In a pivotal phase II study, SAR3419 was associated with an ORR of 44% and a CR rate of 15% in 41 eligible patients with R/R DLBCL who had previously received rituximab-containing immunochemotherapy [80]. Patients who received SAR3419 based on a schedule of 4 weekly doses followed by 4 biweekly administrations had moderate grade 1–2 myelosuppression, ocular toxicity, and neurotoxicity reported at grade 1 to 2. The estimated median OS for all treated patients was 9.2 months, with a median PFS of 4.4 months. The ORR, the primary endpoint, was unfavorable (31%) in 45 patients with R/R DLBCL in a multicenter phase II study of SAR3419 and rituximab [81]. The results were possibly due to the high proportion of patients with primary refractory disease in the trial. The treatment-related toxicities were mainly gastrointestinal symptoms (52%) and asthenia (25%). Although the drug is well tolerated, its development remains challenging because of its undesirable efficacy.

#### Denintuzumab mafodotin

Denintuzumab mafodotin (SGN-CD19A) is an ADC aimed at CD19 formed by conjugation of a potent microtubule-acting agent, MMAF, to a humanized anti-CD19 IgG1 mAb through a non-cleavable linker. In a phase I trial, 33% of patients with R/R B-NHL who received at least one prior salvage therapy responded to denintuzumab mafodotin monotherapy (22% CR), achieving a median DOR of 40 weeks and low incidence rates of hematological disorders and neuropathy [82]. In the subgroup of relapsed patients, promising outcomes were observed in terms of the ORR (60%) and CR rate (40%) as well as a long-lasting response (median DOR, 47 weeks). Of note, reversible ocular disorders such as superficial microcystic keratopathy occurred in 84% of patients. The results of subsequent phase II trials, however, did not confirm therapeutic efficacy. One study (NCT02855359) was terminated because severe hematological AEs occurred in all recruited patients, with two fatal cases reported, which were likely due to severe myelosuppression and infections. Another trial (NCT02592876) was also discontinued based on the similar toxicity (e.g., febrile neutropenia) observed in the denintuzumab mafodotin plus rituximab, ifosfamide, carboplatin, etoposide, and mesna (RICE) arm for participants with DLBCL.

### Anti-CD22 ADCs

CD22, whose expression is restricted to mature B lineage cells, imposes modulatory effects on diverse signaling pathways that allow appropriate B cell homeostasis, survival, and activation [83]. CD22 is a recycling endocytic receptor that shuttles toxins between the cell surface and endosomes, leading to rapid internalization [84]. The anti-CD22-based treatment already provides a path forward to overcome the poor prognosis of R/R B lineage lymphomas.

#### Inotuzumab ozogamicin

InO is composed of a humanized anti-CD22 IgG4 mAb, a hydrazone linker, and a calicheamicin derivative as its payload. It was initially applied in acute lymphoblastic leukemia (ALL) patients in various clinical settings [85–87]. The possibility of InO application in R/R NHL is supported by data from several clinical studies [88–93], which used it in various combinations, such as with rituximab, gemcitabine, dexamethasone, and cisplatin (R-GDP) or rituximab, cyclophosphamide, vincristine, and prednisone (R-CVP) therapy. Rituximab plus InO (R-InO) treatment was associated with ORRs of 87%, 74%, and 20% for relapsed FL, relapsed DLBCL, and refractory aggressive NHL, respectively, in a phase III study (NCT00299494) [88]. The 2-year PFS rate was 68% for relapsed FL and 42% for relapsed DLBCL. Regarding another phase III trial for patients with R/R aggressive NHL, the result was somewhat disappointing because no superior therapeutic efficacy was observed in the R-InO arm compared with rituximab plus chemotherapy with bendamustine or gemcitabine arm (ORR, 41% vs. 43%; OS, 35% vs. 37%) [89].

Based on the undesirable outcomes of R-InO treatment, several novel combination therapies for R/R NHL have emerged recently. A phase I study (NCT01055496) of the InO plus R-GDP regimen demonstrated manageable toxicity and relatively lower required doses in patients with R/R NHL [90]. The ORR was reported to be 53%, with 20% CR and 33% PR. Subset analysis revealed that the ORRs were 71%, 62%, and 33% in patients with FL, DLBCL, and MCL, respectively. The major high-grade (≥ 3) events were thrombocytopenia (75%) and neutropenia (62%). In addition, one phase I trial (NCT01055496) treated patients with R/R B-NHL with InO therapy at 0.8 mg/m^2^ plus standard-dose R-CVP [91]. The ORR was 84%, and the CR rate was 24%. The main toxicity (grade 3) associated with InO in the maximum-tolerated dose (MTD) cohort was hematological, including neutropenia (74%), thrombocytopenia (50%), leukopenia (47%), and lymphopenia (42%). Overall, the toxicity profile of this combination was consistent with those previously reported for InO alone [94] or in combination with rituximab [95]. The R-InO and R-GEMOX combinations are being compared in a phase Ib/II study (NCT01562990) in patients with DLBCL at first or second relapse. The efficacy and safety of InO were also evaluated in a phase II trial (NCT00868608) for patients with indolent B-NHL refractory to rituximab-based therapies [92]. The reported ORR (67%) and CR rate (31%) for all enrolled patients in this setting were promising, with a median PFS of 12.7 months. Treatment duration was restricted by hematological toxicities, particularly thrombocytopenia (74%) and neutropenia (56%). InO in combination with conventional chemotherapy is also tested in different settings. A phase II study (NCT03856216) aims to assess AEs and efficacy of InO plus chemotherapy in participants with lymphoma or leukemia in the pre- and post-transplantation settings.

#### Pinatuzumab vedotin

Pina is an ADC with a humanized anti-CD22 IgG antibody that can be linked to MMAE via an optimized protease-cleavable linker. A phase I study evaluated the feasibility of the therapeutics of R-pina and obtained its recommended phase II dose (RP2D) in R/R DLBCL and R/R indolent NHL [96]. Overall, neutropenia and PN were the most frequent causes of drug withdrawal and were the primary observed DLTs. As a result, 2.4 mg/kg every 3 weeks was designated the RP2D for both Pina alone and Pina with rituximab. With this RP2D, the ORR was 36% in R/R DLBCL and 50% in R/R indolent NHL. Furthermore, the subgroup analysis of DLBCL displayed encouraging outcomes as the ORR and CR rate of R-pina appeared superior to those of the single-agent Pina (ORR, 57% vs. 36%; CR rate, 24% vs. 16%, respectively). According to the results of the phase II ROMULUS study (NCT01691898), the ORR and CR rate in the 21 patients with FL receiving R-pina were 62% and 5%, respectively, and the 42 patients in the DLBCL subgroup had an ORR of 60% and a CR rate of 26% [71]. Severe AEs occurred in 79% of the 42 patients with FL and 62% of the 21 patients with DLBCL. Pina was not chosen for further testing, partly because it was relatively less effective with higher grade AEs.

#### TRPH-222

TRPH-222 (CAT-02-106), a site-specifically conjugated ADC, targets CD22 with a maytansine payload coupled through a novel non-cleavable linker utilizing SMARTag® HIPS bioconjugation technology [97]. The preliminary result of an ongoing phase I clinical trial (NCT03682796) suggested that 5 (22.7%) of 22 heavily pretreated patients were confirmed to have a CR at doses of 0.6–5.6 mg/kg with an overall favorable safety profile. Notably, no severe peripheral neuropathies, which are generally observed with ADCs containing microtubule-interacting payloads, have been observed in the patients to date. Another preclinical study revealed that TRPH-222 may be an efficacious approach for MDR1-resistant cells with no apparent side effects in NHL xenograft animal models when they were repeatedly dosed [98]. Among all tested animals, TRPH-222 induced a CR in 75% (6 of 8) and a durable response at the end of the study in 38% of these animals. Collectively, patients may benefit from this drug, including those who become refractory to standard chemotherapies or resistant to prior immunotherapies, because of the upregulation of MDR proteins.

### Anti-CD20 ADCs

Several second-generation mAbs targeting CD20, a broadly explored target in the DLBCL population, have been clinically tested, but no significant benefits have been shown for mAb therapies over the standard R-CHOP regimen [99]. Hence, a strong rationale exists for examining the roles of CD20-ADCs rather than CD20-mAbs in clinical applications.

#### MT-3724

MT-3724 is an innovative engineered anti-CD20 ADC that contains a variable fragment (scFv) bonded to Shiga-like toxin-1A (SLTA), which expedites both internalization and cell killing by ribosomal inhibition. The results of a phase I trial (NCT02361346) of MT-3724 monotherapy demonstrated clinical anti-neoplastic activity in heavily pretreated R/R B-NHL patients, especially in the advanced-stage DLBCL subset with an ORR of 30% [100]. The most common AEs related to the drug were edema (67%), fatigue (43%), diarrhea (38%), myalgia (38%), and cough (33%). To estimate the feasibility of combining MT-3724 with chemotherapies or immunomodulatory agents, MT-3724 was tested in combination with Len, revealing potentiated cytotoxicity in CD20-positive cell lines and supporting the further assessment of MT-3724 in a phase II trial in combination with Len in adult patients with R/R NHL [101].

### Anti-CD25 ADCs

CD25 is the α subunit of the IL-2 receptor (IL-2Rα) and can increase the affinity of ligand binding and active oncogenic signaling pathways [102]. Indeed, upregulated CD25 expression is related to a more aggressive course and an inferior outcome in DLBCL, FL, HL, and CLL [103–106]. It has also been shown to promote lymphomagenesis and drug resistance in T cell lymphomas [107].

#### Camidanlumab tesirine

Camidanlumab tesirine (ADCT-301) is an ADC comprising human IgG1 that is stochastically coupled with a protease-cleavable linker and has a PBD dimer as its payload [108]. The drug was evaluated in R/R NHL and R/R cHL in a phase I trial (NCT02432235), showing a promising antitumor activity profile, especially cHL and the T cell lymphoma subtype [108, 109]. In the cHL group, the ORR was 69%, with 44% CR. Moreover, the ORR and CR rate were 81% and 50%, respectively, in a population of 26 heavily pretreated patients in the 45 µg/kg dose group. The trial also reported a superior ORR of 50% in the T cell lymphoma subgroup versus an ORR of 31% in the B cell lymphoma subgroup. Further analysis based on prior therapies showed an ORR of 81% for patients with cHL who previously received BV. The median DOR and PFS were 7.7 and 6.7 months in the cHL group, respectively. Common TEAEs comprised fatigue (42%), rash (33%), liver abnormality (30%), and pyrexia (30%), and 16 (27%) cases of peripheral edema or effusion were also noted. Although it is still under investigation, ADCT-301 may be a potential therapeutic option for patients with HL after failure to BV.

### Anti-CD30 ADCs

Anti-CD30-LDM, an innovative ADC with an intact anti-CD30 antibody conjugated to LDM via a non-cleavable linker, presents attractive tumor-targeting capability and antitumor efficacy both in vitro and in vivo [110]. Treatment with anti-CD30-LDM remarkably inhibited tumor growth in mice in a dose-dependent manner without apparent AEs. Anti-CD30-LDM exerts its cytotoxic effects by inducing DNA damage instead of by blocking the polymerization of tubulin, highlighting its potential for overcoming resistance induced by BV. Additionally, anti-CD30-LDM augmented programmed cell death-1 ligand 1 (PD-L1) presentation in cell lines, indicating possible antitumor synergy between anti-CD30-LDM and immunotherapy agents. Consequently, it could be chosen for further testing as a single agent or combination therapy.

### Anti-CD37 ADCs

CD37 is a member of the tetra-spanning superfamily, which directly mediates survival and apoptotic signaling, and is expressed on normal and malignant mature B cells, similar to CD20. CD37 has been introduced as an appealing target for treating DLBCL because the expression level  of CD37 on neoplastic cells in patients with DLBCL correlates with PFS and OS [111]. To date, the development of anti-CD37 ADCs has shown limited progression due to limited clinical efficacy.

#### Naratuximab emtansine

Naratuximab emtansine (also known as IMGN529) is a CD37-directed ADC consisting of a humanized IgG1 mAb linked to DM1 via succinimidyl-4-(*N*-maleimidomethyl)-cyclohexane-1-carboxylate (SMCC), a thioether linker [112]. A pivotal phase I trial reported that the MTD and RP2D of IMGN529 were 1.4 mg/kg and 0.7 mg/kg, respectively, in adult patients with R/R B-NHL [113]. Among 39 response-evaluable patients, the efficacy was limited, with an ORR of 13% (1 CR and 4 PRs, 4 of which occurred in the DLBCL subset). The most frequent TEAEs were fatigue (39%), neutropenia (37%), thrombocytopenia (37%), and pyrexia (37%). Additional preclinical studies revealed that the combination of IMGN529 with rituximab had potentially enhanced antitumor efficacy compared with either single agent [114]. At present, a phase II trial (NCT02564744) is ongoing to investigate the combination of these two agents in a larger cohort.

#### AGS67E

A humanized anti-CD37 IgG2 mAb binds to MMAE via a protease-cleavable linker, and the resulting ADC, AGS67E, retains the inherent antibody activities. The intrinsic effects of AGS67E include direct proapoptotic activity and potent cytotoxicity, leading to cell death in several types of NHL and AML cell lines [115]. First, a phase I trial (NCT02175433) described a moderate response in terms of an ORR of 22% and CR rate of 14% in 50 patients with R/R B-NHL and R/R T-NHL, and a small number of patients experienced PN (16%) and neutropenia (8%) [116]. One ongoing, multicenter phase I study enrolled 30 participants who had received at least two prior systemic therapies treated with AGS67E and determined the MTD with or without G-CSF [117]. Specifically, the preliminary results reported that two subjects with DLBCL achieved a CR, and two subjects (one with MF and one with DLBCL) achieved a PR. The neutropenia that occurred in the trial may largely be attributed to the bystander effect, and G-CSF administration appeared to significantly improve the recovery rate [118]. Expansion cohorts are planned at the MTD with or without G-CSF.

### Anti-CD70 ADCs

CD70 is a member of the TNF receptor superfamily and acts as a functional receptor binding to soluble CD27. CD70 is expressed on some types of lymphomas and solid tumors, where it correlates with poor prognosis [119, 120]. Three anti-CD70 ADCs had reached phase I clinical development in patients with CD70-positive R/R B-NHL and metastatic renal cell carcinoma (RCC). Unfortunately, none of these ADCs was retained for further evaluation due to a lack of clinical benefit and excess toxicity.

SGN-CD70A, an ADC, contains an antibody against CD70 linked to the PBD dimer via a protease-cleavable linker. A first-in-human phase I study described early-onset severe side effects and undesirable efficacy in patients with R/R CD70-positive NHL, including DLBCL, MCL, and grade 3b FL [121]. The application of SGN-CD70A has been limited due to the occurrence of grade 4 thrombocytopenia, albeit with long-lasting remission. MDX-1203 (BMS-936561) comprises a fully human IgG1 antibody specific for CD70 linked through a protease-cleavable linker attached to a duocarmycin derivative. The dose-escalation phase I trial was discontinued on account of limited antitumor activity for patients who received more than three prior systemic administrations [122]. Vorsetuzumab mafodotin (SGN-75) is a humanized anti-CD70 ADC with an IgG1 mAb, MMAF payload, and a non-cleavable linker. Given its unacceptable toxicity (idiopathic thrombocytopenic purpura), a phase I study in patients with R/R CD70-positive NHL or metastatic RCC was terminated [123]. Considering that alleviating thrombocytopenia is currently not possible, the applicability of CD70-targeting ADCs remains limited.

### Anti-CD74 ADCs

CD74 is a type II transmembrane glycoprotein that is involved in the presentation of endogenous antigens and the process of immune regulation. The expression of CD74 protein is found in a wide variety of normal tissues and lymphoid malignancies, supporting the application of anti-CD74 antibodies for targeted immunomodulatory therapies [124].

Only 2 CD74-targeting ADCs (STRO-001 and hLL1-DOX) have reached clinical development. STRO-001 is a humanized and glycosylated anti-CD74 ADC that is generated by novel cell-free protein synthesis technology and integrates a non-cleavable linker and maytansinoid payload. It exhibits a high degree of homogeneity and stability because of its site-specific conjugation. Preliminary data from an open-label phase I study (NCT03424603) of STRO-001 in heavily pretreated patients with advanced B cell malignancies provided early signs of efficacy and a good safety profile [125]. The ORR was 25% in 16 evaluable patients with no ocular or neuropathy disorders observed. The study recently began to dose patients at planned levels of 2.5 mg/kg and 3.5 mg/kg. Milatuzumab doxorubicin (hLL1-DOX) was also explored in a phase I/II trial (NCT01585688) of patients with R/R B-NHL or CLL, but unsatisfactory results led to trial discontinuation [126]. SP7676, another anti-CD74 ADC, has a high degree of stability in circulation via site-specific conjugation. It elicits a strong response, as evidenced by 100% of animals achieving complete regression of tumors in lymphoma models, and SP7676 achieved the intended pharmacodynamic effect and produced tumor regression in the "double-hit" lymphoma models [127]. Nevertheless, the development of these drugs is still in the early stage, and further exploration is needed to ascertain their value in clinical applications.

### Anti-CD79b ADCs

#### Iladatuzumab vedotin

In addition to Pola, iladatuzumab vedotin (DCDS0780A), another ADC directed against CD79b featuring a protease-cleavable linker and MMAE payload, has been introduced. A first-in-human phase I trial (NCT02453087) recently described the preliminary outcomes of iladatuzumab vedotin alone or in combination with rituximab in patients with R/R B-NHL or CLL. One primary endpoint, improvement in ORR, was not satisfactorily met because the ORR was only slightly greater for the rituximab combination group (85% vs. 84%) [128]. Among all patients with DLBCL (*n* = 24), the ORR was 60% (43% CR). In a pooled patient population, 43% experienced ocular disorders leading to dose reduction or drug interruption, and 22% experienced neutropenia (grade ≥ 3). The unsatisfactory results and ocular toxicity observed have hindered the progress of iladatuzumab vedotin into phase II trials.

### Anti-ROR1 ADCs

ROR1 is an oncofetal glycoprotein expressed on CLL, MCL, a variety of malignant tumors, and early-stage B cells but is rarely expressed in normal adult tissues. Moreover, a high level of ROR1 expression in cancer cells appears to be involved in the inhibition of apoptosis and predicts an inferior outcome in primary refractory DLBCL, low-grade FL, and Richter´s syndrome [129].

#### VLS-101

VLS-101, an innovative ADC, comprises a UC-961 linker, MMAE, and an anti-ROR1 receptor. VLS-101 caused almost complete tumor regression, which was observed even in models without universal ROR1 expression, suggesting MMAE bystander killing [130]. It also induced a long-term response and considerably extended survival after treatment discontinuation. A phase I clinical trial (NCT03833180) of VLS-101 investigated patients who failed a median of 4 prior systemic therapies with MCL (*n* = 15), CLL (*n* = 7), DLBCL (*n* = 5), FL (*n* = 3), or MZL (*n* = 1) [131, 132]. The reported ORR was 47%, with 20% of MCL patients achieving a CR. In the DLBCL subgroup, an ORR of 80% (40% CR) was seen in 5 patients. Among 15 patients with R/R MCL, 100% of patients received BTK inhibitors, including 87% of patients who progressed on BTK inhibitors. Grade 4 neutropenia occurred in 28% of patients, with 3% of patients having neutropenic fever. Considering the data above, VLS-101 had signs of potential clinical benefits in aggressive DLBCL or MCL with tolerable side effects in the study. Phase I/II studies of VLS-101 monotherapy and combination therapy in patients with hematological tumors are currently under investigation.

#### Cirmtuzumab-ADC-7

Cirmtuzumab-ADC-7, another ADC including a humanized IgG1 mAb and a cirmtuzumab (UC-961)-linker connecting MMAE, was selected for further testing in CLL and MCL cell lines because it was well tolerated with high stability and signs of efficacy in preclinical studies [133]. In addition, drugs such as ibrutinib that inhibit the BCR pathway can impair ROR1 expression and consequently ROR1-targeting treatments, suggesting a potential role for investigating combination therapeutics [134]. Cirmtuzumab-ADC-7 plus venetoclax (a BCL2 antagonist) caused almost complete regression of tumors in xenograft models, indicating the potent synergistic cytotoxicity of combining these two agents. Taken together, cirmtuzumab-based ADCs have promise and warrant further investigation for the treatment of patients with ROR1-positive malignancies.

### ADCs targeting other antigens

ADCs under preclinical exploration and demonstrating activity in animal models of lymphomas include those targeting several antigens that have not been described in the aforementioned parts (CD38, CD123, CD185, CD205, and HLA-DR) (Table 2).

**Table 2:** Preclinical antibody–drug conjugates active in animal models of lymphoma

Agent	Target	Linker	Payload	Indication
huB4-DGN462	CD19	Sulfo-SPDB	DGN462	B cell lymphoma [150]
RC58-based ADC	CD19	Maleimide-PEG-based linkers	/	B cell lymphoma [151]
Anti-CD30-LDM	CD30	Non-cleavable linker	LDM	HL and ALCL [110]
CD38-077	CD38	Non-polyethylene glycol linker	Duostatin 5.2	Burkitt's lymphoma and MM [152]
BAY-943 (IL3RA-ADC)	CD123	Protease-cleavable linker	KSP inhibitor	AML and HL [153]
BAY 924	CD185	/	KSP inhibitor	B cell lymphoma [154]
MEN1309/OBT076	CD205	SPDB	DM4	B cell lymphoma [155]
IMMU-140	HLA-DR	Cleavable linker	SN-38	HL, NHL and CLL [156]

ALCL, anaplastic large cell lymphoma; HL, Hodgkin lymphoma; MM, multiple myeloma; AML, acute myeloid leukemia; NHL, non-Hodgkin lymphoma; CLL, chronic lymphocytic leukemia; KSP, kinesin spindle protein

## Future perspectives

In the modern era, ADCs in combination with other potent antitumor drugs have been proposed to achieve a durable response and to broaden the therapeutic window of combination agents. Based on the encouraging outcomes of several clinical trials, studies on ADCs have assessed their roles in the first-line, consolidation, and salvage settings. Notably, combination treatment, such as ADCs in combination with immune checkpoint blockades, may provide synergistic clinical activity. However, accompanying toxicity may occur [135–138]. To improve the response rates, it is important to select novel targets that are exclusively expressed on cancer cells. Progress has been achieved in designing novel antibody formats to optimize the stability and DAR of ADCs, which may facilitate tumor uptake. Efforts have also been made to apply novel kinds of linkers in ADCs to augment the bystander effect and thereby target heterogeneous tumors or cancers with homogeneous but low target expression. Given the accumulation of many incremental advances in technology, such as site-specific conjugation approaches, the generation of highly efficacious and less toxic ADCs has become possible.

When patients show undesirable responses or are insensitive to ADCs, opportunities also exist to innovate in ADC payloads, moving beyond typical cytotoxic drugs to immunotherapeutic agents rationally chosen for their antitumor activity. Novel ADCs may employ NAMPT inhibitors as a new nonantimitotic payload targeting T cell lymphomas [139]. It is worth noting that ZW38 is a novel bispecific ADC engineered to induce increased response rates, reduce the incidence of relapse, and ameliorate the therapeutic index [140].

Likewise, advances in biological platforms have paved the way to expanding the possibilities for ADC development. For example, THIOMAB, a novel cysteine engineered technology, was proposed to optimize the bioconjugation efficiency and allow the generation of more stable and efficacious ADCs with controlled DARs, thereby generating less toxic ADCs [141, 142]. A high-throughput conjugation strategy was developed for THIOMAB antibodies to identify the sites suitable for linking to auristatin derivatives [143]. Additionally, cell-free protein synthesis has emerged as an attractive candidate to produce more homogenous ADCs by introducing payloads at one or more defined sites, thus developing more stable and less toxic ADCs [144]. A gene engineering technology, the CRISPR/Cas-9 system, offers the opportunity to engineer Fab molecules and allows for dual site-specific conjugation without compromising target affinity [145]. Harnessing the platform to produce Fab fragments and be equipped with two distinct cytotoxic payloads appears to be a promising option to obtain a better therapeutic outcome while reducing the toxicity of the combination therapy.

The expanding repertoire of applicable ADCs in the clinic has also raised interest in the issue of ADC resistance. Studies to date have implied some potentially causative factors, including downregulation of the antigen, mutation of the antigen, overexpression of drug transporters, variations in ADC distribution, and alteration of the lysosomal environment [146]. One study revealed that acquired resistance to PBD dimer-containing ADCs may be attributed to specific ATP-binding cassette drug transporters, which may guide decision-making in drug combination strategies [147]. Further investigation could focus on non-internalizing ADCs targeting the tumor microenvironment to avoid ADC resistance. Moreover, new technologies such as single-cell sequencing could be used in the development of more accurate biomarker assessments that can avoid the interference of tumor heterogeneity and contribute to overcoming drug resistance [148, 149].

## Conclusion

ADCs are a promising treatment that has risen to prominence over the past three decades. Antibodies, linkers, and payloads, every aspect of ADCs, are evolving dramatically and will be fundamental to optimizing the design and further development of this therapeutic class. Newly developed ADCs are more strategically designed compounds and benefit from improvements in tumor specificity, off-target toxicity, and payload potency. “Old” challenges, such as drug resistance, will probably be resolved by lymphoma therapeutics leveraging ADCs in combination with other agents. With the emergence of enormous numbers of combinatorial approaches, there is an urgent need for robust phase I testing to ensure manageable toxicity and plan feasible combination-dosing regimens. In addition, innovative biomarker measurements and appropriate patient selection strategies will pave the way for ADC development.

## Data Availability

Not applicable.

## Abbreviations

ADC: Antibody-drug conjugate
mAb: Monoclonal antibody
IgG: Immunoglobulin G
ADCC: Antibody-dependent cytotoxicity
ADCP: Antibody-dependent cell-mediated phagocytosis
DAR: Drug-to-antibody ratio
PBD: Pyrrolobenzodiazepine
MMAE: Monomethyl auristatin E
MMAF: Monomethyl auristatin F
DLT: Dose-limiting toxicity
DM1: N20-deacetyl-N20-(3-mercapto-1-oxopropyl)-maytansine
DM4: N20-deacetyl-N20-(4-mercapto-4-methyl-1-oxopentyl)-maytansine
NHL: Non-Hodgkin lymphoma
KSP: Kinesin spindle protein
SPDB: *N*-Succinimidyl-4-(2-pyridylthio)-butanoate
SPP: *N*-Succinimidyl-4-(2-pyridyldithio)-pentanoate
Ala: Alanine
Cit: Citrulline
Val: Valine
MC: Maleimidocaproyl
SMCC: Succinimidyl-4-(*N*-maleimidomethyl)-cyclohexane-1-carboxylate
BV: Brentuximab vedotin
Pola: Polatuzumab vedotin
Pina: Pinatuzumab vedotin
HL: Hodgkin lymphoma
cHL: Classical Hodgkin lymphoma
ALCL: Anaplastic large cell lymphoma
PMBCL: Primary mediastinal B cell lymphoma
ASCT: Autologous stem cell transplantation
HSCT: Hematopoietic stem cell transplantation
Len: Lenalidomide
Nivo: Nivolumab
ORR: Overall response rate
CR: Complete response
PR: Partial response
OS: Overall survival
PFS: Progression-free survival
R/R: Relapsed or refractory
AE: Adverse event
PN: Peripheral neuropathy
PTCL: Peripheral T cell lymphoma
CTCL: Cutaneous T cell lymphoma
DLBCL: Diffuse large B cell lymphoma
DOR: Duration of response
FL: Follicular lymphoma
MCL: Mantle cell lymphoma
G-CSF: Granulocyte colony stimulating factor
TEAE: Treatment-emergent adverse event
RP2D: Recommended phase II dose
MTD: Maximum-tolerated dose
